# Influences of Modified Sm_2_O_3_ on Thermal Stability, Mechanical and Neutron Shielding Properties of Aminophenol Trifunctional Epoxy Resin

**DOI:** 10.3390/polym14030638

**Published:** 2022-02-08

**Authors:** Hongqing Wang, Qunying Huang, Yutao Zhai

**Affiliations:** 1 Institutes of Physics Science and Information Technology, Anhui University, Hefei 230601, China; 2 Institute of Nuclear Energy Safety Technology, Hefei Institutes of Physical Science, Chinese Academy of Sciences, Hefei 230031, China; qunying.huang@inest.cas.cn (Q.H.); yutao.zhai@inest.cas.cn (Y.Z.)

**Keywords:** aminophenol trifunctional epoxy resin, samarium oxide, thermal energy neutron source, thermal stability, mechanical property

## Abstract

The requirements regarding the weight and capacity reduction of neutron shielding materials have become an urgent issue for advanced nuclear facilities and plants. An epoxy-based neutron shielding material with high-temperature stability and good neutron irradiation resistance was designed in this paper to solve the above issue. Aminophenol trifunctional epoxy resin (AFG-90H) was compounded with samarium oxide (Sm_2_O_3_) by means of an ultrasonic-assisted method and the compatibility of Sm_2_O_3_ with the AFG-90H matrix was improved by 3-aminopropyltriethoxysilane (APTES) surface modification. Fabricated Sm_2_O_3_-APTES/AFG-90H composites exhibited improved thermal stability, glass transition temperature and Young’s modulus with increased Sm_2_O_3_-APTES content. Neutronics calculation results show that the neutron permeability of 2 mm-thick 30 wt% Sm_2_O_3_-APTES/AFG-90H was 98.9% higher than that of the AFG-90H matrix under the irradiation of the thermal neutron source. The results show that the proper addition range of Sm_2_O_3_-APTES is between 20% and 25%. The Sm_2_O_3_-APTES/AFG-90H composite is a promising neutron shielding material for advanced nuclear system.

## 1. Introduction

Neutron radiation exists not only in nuclear reactors, but also in various industrial, aviation and radiotherapy fields [1,2,3,4]. Uncharged neutrons can easily penetrate human tissues and cause ionization, which seriously threatens human health. Moreover, neutron radiation can also have a fatal negative impact on the service lifetime of electronic components and the environment [5]. As a result, the R&D on radiation protection materials is widely performed in the nuclear-related fields, including neutron shielding materials.

Neutrons shielding processes include two steps: moderating and absorbing [6,7]. High-energy neutrons (>1 MeV) first undergo inelastic scattering with substances with high atomic numbers and then lose energy, or undergo multiple elastic scattering with materials rich in hydrogen and then reduce energy to thermal neutrons (~eV). Thermal neutrons are easily captured or absorbed by elements with high neutron absorption cross-sections [8]. As described above, traditional shielding materials are roughly divided into metal, concrete and polymer materials according to their compositions. At present, the commercial metal-based shielding materials mainly include boron-containing stainless steel. Divya et al. pointed out that the serious problem with boron-containing stainless steels is the formation of low-melting point eutectics, makes this class of steel susceptible to heat cracking [9]. Maurya et al. studied the influence of various processing parameters on the strength, hardness and strain to failure of B_4_C particle reinforced Al-alloy matrix composites. When the content of B_4_C exceeded 10%, the strength and strain of B_4_C/Al to failure decreased due to particle agglomeration [10]. In addition, the commonly used concrete shielding materials mainly include boron-containing concrete and cement composites. Chidiac et al. studied the heat evolution, compressive strength and neutron shielding capabilities of hydrated mixes containing 0, 25, 50 and 75 wt% of B_4_C. The results show that a concrete mix containing up to 50 wt% B_4_C was found to be beneficial to the development of concrete properties and to effective neutron shielding [11]. Sevim et al. studied the strength and fresh properties of borogypsum concrete. They showed that an addition of 4 wt% of boron sludge could reduce the 90-day compressive strength of concrete by 65 wt% [12]. However, the requirements regarding the above materials in nuclear power stations and facilities are a large engineering amount and a heavy weight [13]. There are limitations in transportation and small modular manufacturing. Currently, the fourth-generation advanced nuclear power system puts forward the target of “integration of structure and function” with the demand for weight and capacity reduction. Therefore, developing new neutron shielding materials with outstanding properties has become one of the promoted ways to satisfy the requirements of neutron protection.

Blending hydrogen-rich polymer matrix with high neutron cross-section absorbing elements can integrate neutron moderation and absorption. So, these materials are expected to become alternative materials for metals and concrete in nuclear facilities, such as polyethylene (PE) [14,15], high-density polyethylene (HDPE) [16,17], epoxy resin (EP) [18,19], ethylene propylene diene monomer (EPDM) [20] and polyimide (PI) [21,22], etc. Although PE and EPDM matrix have good processability, they have low thermal stability and it is difficult to use them at a temperature higher than 100 °C for a long time. PI has high temperature stability and good mechanical properties. Wu et al. studied composites based on carbon fiber-reinforced carborane-containing polyimides. The results show that the thermoset polyimide matrixes exhibited high thermal stability with T_5_ > 800 °C both in nitrogen and air environments [23]. However, the synthesis process of PI is more complex than other polymers and it is mostly used in membrane products. It is difficult to break through to large-scale preparation. Among the above polymers, EP has good chemical stability and structure stability. Jiang et al. blended B_4_C particles with AFG-90H to develop a new type of AFG-90H epoxy resin-based composite, and the results show good neutron shielding and mechanical performance at high temperatures [24]. Moreover, owing to the aromatic ring in the main chain of EP, it is mechanically stable under long-term irradiation [25]. Therefore, a new type of three functional epoxy resin AFG-90H was synthesized as the matrix of a neutron shielding material. Compared with ordinary EP, it has better neutron irradiation resistance, mechanical stability and thermal stability [26].

**Figure 1:**
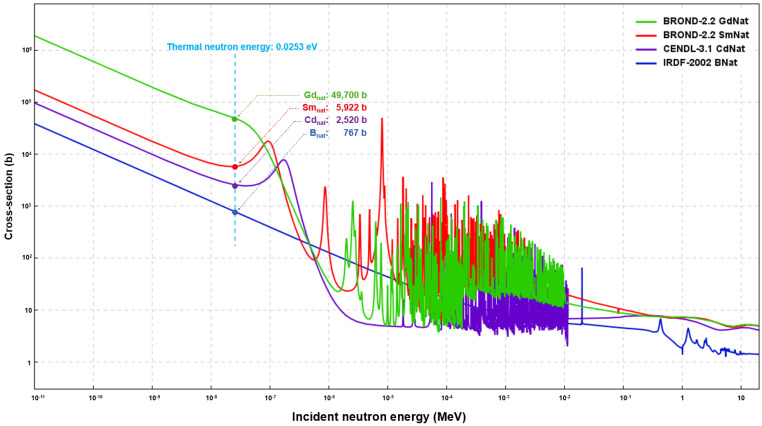
The neutron absorption cross-sections of Gd, B, Cd and Sm.

In order to improve the thermal neutron absorption performance of EP, it is important to select suitable neutron absorbers. The average macro-absorption cross-sections (∑) of the neutron-absorbing elements Gd, B, Cd and Sm are 49,700, 3835, 2520 and 5922 barns at thermal neutron energy, respectively [27]. Figure 1 shows the absorption cross-sections of these four elements at different neutron energies. Gd has the highest thermal neutron capture cross-section among all of the stable nuclei. However, the neutron capture reaction for ^157^Gd and ^155^Gd emits a *γ*-ray cascade with a total energy of ~8 MeV [28]. B and its compounds (e.g., B_4_C and BN) are good thermal neutron absorbers and are widely used in neutron shielding materials. Moreover, B compounds absorb neutrons through the reaction ^10^B(n,α)^7^Li with the concomitant generation of helium bubbles [29]. Such helium bubbles cause the premature failure of materials. As is known, Cd is toxic, carcinogenic and harmful to human health and is not suitable to be used as a radiation protection element. It is usually used as an element in the Ag-In-Cd control rods of reactors [30].

Samarium is found in many natural minerals, such as bastnaesite, monazite and samarskite [31]. The total reserve of Sm in the earth is approximately 2 million tons [32]. Sm is most commonly sold in the form of Sm_2_O_3_, which is one of the cheapest rare earth oxides on the market (USD ~40/kg in China) [33]. Additionally, Sm is also a suitable neutron absorber; the natural abundance of ^149^Sm is 13.9%, and most of its neutron capturing and decay products are also other isotopes of samarium [34]. Sm has a wider neutron absorption spectrum of 10^−7^–10^−5^ MeV compared to Gd and B. Therefore, it has the potential to be used as a neutron absorber for application in shielding materials.

The high content of fillers can cause their uneven distribution in the polymer matrix and poor interface interaction. This defect reduces the mechanical stability of EP composites. One issue is that high neutron shielding and mechanical performances cannot be well balanced. Generally, this is due to large differences in physicochemical properties between the inorganic filler and the epoxy resin matrix [35]. Constructing a chemical bond “bridge” is the key to strengthening interfacial interactions. This “bridge” can enable the orderly grafting of inorganic particles and organic molecular chains to improve the dispersion of fillers in the resin matrix [36]. Covalent modification is a simple fabrication method for particles. The silane coupling agent APTES contains two different chemical functional groups. One end of the functional group chain can react with silanol groups and then connect to the surface of inorganic materials such as glass fiber, silicate and metal oxide, etc. The other end can form covalent bonds with resin molecular chains. Thus, the coupling of the two incompatible materials can realize the equivalent function of a “bridge”.

In this work, Sm_2_O_3_/AFG-90H composites were prepared by solution casting and ultrasonic mixing of epoxy resin matrix (AFG-90H) and surface-modified Sm_2_O_3_. The Sm_2_O_3_ was used as the neutron absorber. Synchronous thermal analysis (TG-DSC) was used to analyze the thermal stability and glass transition temperature of the materials. The integrated neutron transport and safety evaluation software SuperMC [37] was used to analyze the neutron shielding performance of the materials coupled with the data library HENDL1.0 [38]. The microstructure and Fourier-transform infrared (FT-IR) spectroscopy of the Sm_2_O_3_-APTES and Sm_2_O_3_-APTES/AFG-90H were analyzed. The composites were characterized by X-ray diffraction (XRD). The influence of the mass fraction of fillers in the matrix on the mechanical properties of materials was studied.

## 2. Experimental

### 2.1. Materials

AFG-90H (Meisu Plastic Technology Co., Ltd., Shanghai, China.) was selected as the matrix, with 4,4′-diaminodiphenylsulfone (Yinsheng Chemicals Co., Ltd., Suzhou, China.) as the curing agent. Samarium oxide (Sm_2_O_3_, purity 99.9%), liquid γ-aminopropyl triethoxysilane (APTES, 97%) and toluene (40%) were purchased from Runxiang Chemical Co., Ltd. (Changzhou, China) and Aladdin (Shanghai, China), respectively. Acetone and anhydrous ethanol were acquired from Sinopharm Chemical Reagent Co., Ltd. (Shanghai, China). Deionized water was produced by a Kertone labVIP (Hengqing Technology Co., Ltd., Hangzhou, China) machine and used in all experimental procedures. All chemical reagents and solvents were used as received. The molecular weight, density, crosslinking density, viscosity and epoxy equivalent of the AFG-90H resin are listed in Table 1.

### 2.2. Surface Modification of Sm_2_O_3_ Powder

**Figure 2:**
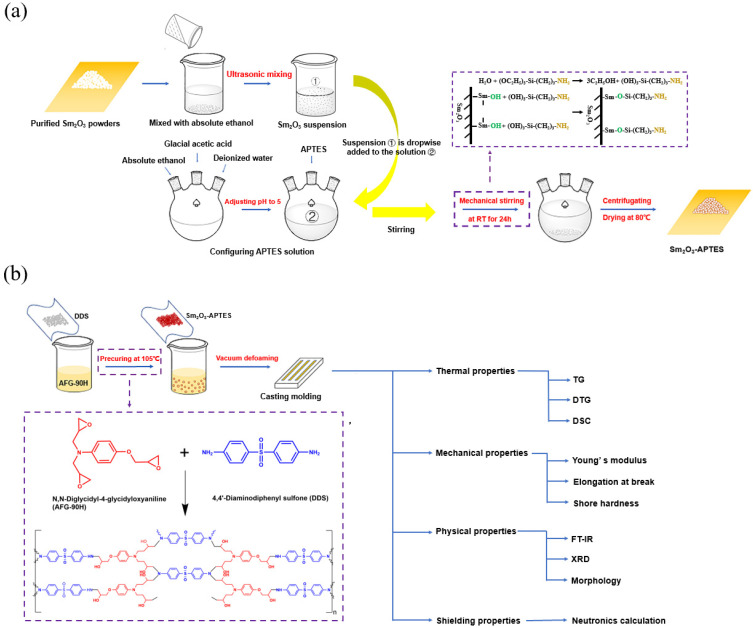
Schematic diagrams of modification process and curing progress: (**a**) scheme of the procedure for the preparation of Sm_2_O_3_-APTES; (**b**) diagram of curing reaction of *N*,*N*-diglycidyl-4-glycidyloxyaniline and 4,4′-aminodiphenyl sulfone.

The silane coupling agent (APTES) was employed to modify the interfacial interaction of Sm_2_O_3_ particles. First, Sm_2_O_3_ particles were dispersed in 60 mL of toluene and stirred under nitrogen gas environment for 20 min before drying. This process was undertaken to clean the surface of the Sm_2_O_3_ particles. Secondly, Sm_2_O_3_ particles were ultrasonicated in 50 mL absolute ethanol for 40 min to form hydroxyl groups on the surface of the Sm_2_O_3_ particles. Absolute ethanol, deionized water and APTES were prepared as a solution with a volume ratio of 9:1:2.5. The glacial acetic acid was used to adjust the pH to 5. The hydrolyzed APTES solution was obtained after full stirring. Under mechanical stirring, the hydrolyzed APTES solution was added dropwise to the Sm_2_O_3_ suspension to react at room temperature for 24 h. After centrifuging the suspension, the particles were cleaned with absolute ethanol and acetone successively. Finally, the clean Sm_2_O_3_ powders were put into the oven and dried for 5 h. The surface modification procedure is shown in Figure 2a.

### 2.3. Fabrication of Sm_2_O_3_-APTES/AFG-90H

To prepare Sm_2_O_3_-APTES/AFG-90H composites, different contents of Sm_2_O_3_-APTES (0–30 wt%) were dispersed into ethanol and ultrasonicated to form a uniform suspension. Subsequently, DDS and AFG-90H were configured into a pre-cured resin at a weight ratio of 5.58:10 and stirred in an oil bath at 105 °C to a low-viscosity state. Then, the dried Sm_2_O_3_-APTES particles were poured into the resin solution under mechanical stirring and ultrasonically treated for 1 h to ensure that the Sm_2_O_3_-APTES was evenly dispersed in the resin matrix. The mixed resin solution was dried at 80 °C to remove ethanol. Finally, the stirred mixture was poured into a Teflon mold and degassed at 90 °C to remove air bubbles. The Sm_2_O_3_/AFG-90H composites was cured at 185 °C for 3 h and then removed from the mold. The diagram of the curing reaction is shown in Figure 2b.

### 2.4. Characterization Methods

Fourier transform infrared (FT-IR) spectra were measured using a Nicolet iS 10 spectrometer with the KBr squash technique and the test device was used to characterize the peaks of functional groups. X-ray diffraction (XRD) analyzed on the AFG-90H and Sm_2_O_3_ particles were performed on Rigaku Ultima IV X-ray diffractometer from 10° to 80°. Thermogravimetric analysis (TGA) and differential scanning calorimetry (DSC) were performed in an air atmosphere using a NETZSCH 449 F3 synchronous thermal analyzer under the heating rate of 10 °C/min. TG-DSC was used to analyze the thermal stability and glass transition temperature of Sm_2_O_3_-APTES/AFG-90H. Scanning electron microscopy (SEM, Carl Zeiss AG, Oberkochen, Germany) was performed to characterize the morphology of Sm_2_O_3_ particles and the surface of Sm_2_O_3_/AFG-90H composites, including its tensile cross-section of the tensile fracture test. Static mechanical testing was performed in accordance with ASTM D638-91 using an Instron 3369 Universal Testing Machine with a loading rate of 2 mm/min. At least four separate dumbbell-shaped samples were tested for each experiment. The Young’s modulus (E) and elongation at break (e) of all samples were tested via the Instron 3369 machine. The Shore hardness of specimens was measured using a Shore D durometer in accordance with ASTM D2240 Shore hardness test method. The Shore hardness was measured five times at the surface with an interval of at least 6 mm for each sample and the average values were calculated.

## 3. Results and Discussion

### 3.1. Surface Modification of Sm_2_O_3_ Powders

**Figure 3:**
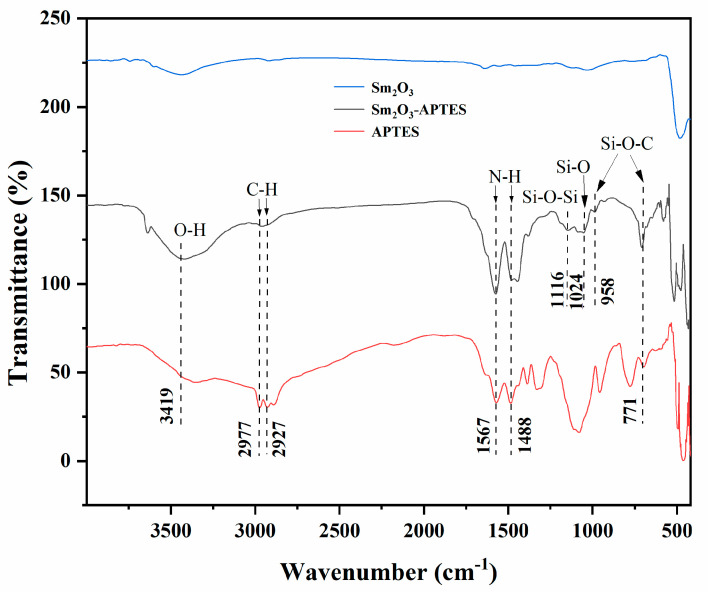
FT-IR spectra of APTES, Sm_2_O_3_ and Sm_2_O_3_-APTES.

**Figure 4:**
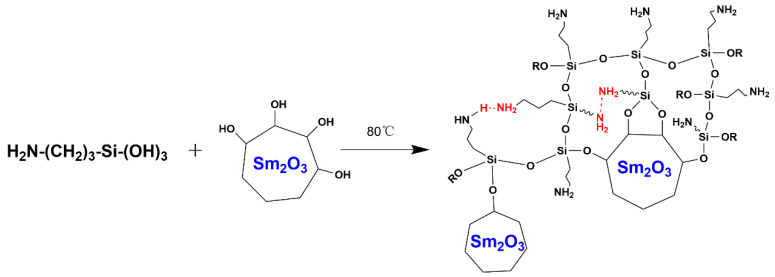
Functional mechanism of APTES on Sm_2_O_3_ powders.

The infrared spectra of APTES, Sm_2_O_3_ and Sm_2_O_3_-APTES are shown in Figure 3. As shown in the FT-IR spectrum of APTES, the absorption peak of the stretching vibration mode of O-H is 3419 cm^−1^. The antisymmetric stretching vibration mode peaks of C-H are 2977 and 2927 cm^−1^. Furthermore, the characteristic peaks of Si-O-C are 958 and 771 cm^−1^. The in-plane bending vibration mode peaks of N-H are 1488 and 1567 cm^−1^. The characteristic peaks of Si-O-Si and Si-O are 1116 and 1024 cm^−1^ [39]. The Si-O-Si bond formed on the surface of non-Si-based materials was a weak chemical interaction formed between two APTES molecules [40]. When comparing the FTIR spectrums of APTES and Sm_2_O_3_-APTES, no obvious peaks were observed in the original Sm_2_O_3_ powders. In contrast, as shown in the FTIR spectrum of Sm_2_O_3_-APTES, all types of characteristic peaks of N-H at 1567 and 1488 cm^−1^ were more prominent than in the APTES spectrum. This indicated that more Sm_2_O_3_-APTES particles were connected to each other through N-H bonds (as shown in Figure 4). In addition, the weak N-N characteristic peak can be seen at 1372 cm^−1^. The FT-IR results indicate that the silane groups in APTES successfully formed covalent bonding with the surface of Sm_2_O_3_.

### 3.2. XRD Patterns of Sm_2_O_3_ and Sm_2_O_3_-APTES/AFG-90H Composites

**Figure 5:**
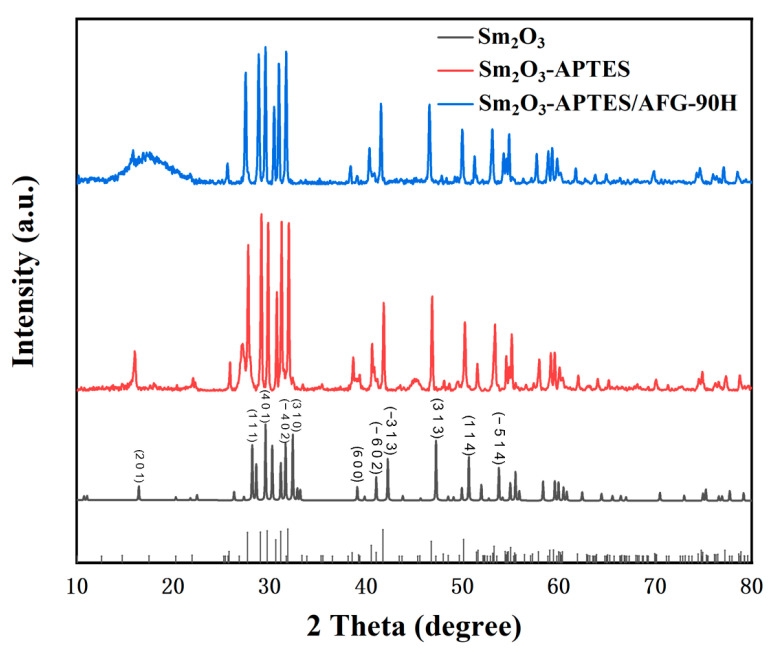
XRD patterns of Sm_2_O_3_, Sm_2_O_3_-APTES and Sm_2_O_3_-APTES/AFG-90H.

The XRD diffraction patterns of Sm_2_O_3_, Sm_2_O_3_-APTES and Sm_2_O_3_-APTES/AFG-90H are shown in Figure 5. The multimodal diffraction pattern of Sm_2_O_3_ showed that it belonged to the monoclinic crystal system. In addition, the Sm_2_O_3_-APTES showed a similar trend as the original Sm_2_O_3_, indicating that the crystal structures of Sm_2_O_3_ did not change during the surface modification process of APTES. Obviously, the existence of the characteristic signal of Sm_2_O_3_ at 2θ = 25–65° in the XRD pattern of Sm_2_O_3_/AFG-90H composites confirmed the successful incorporation of Sm_2_O_3_-APTES into the epoxy resin. The Sm_2_O_3_-APTES/AFG-90H exhibits a broad hump at approximately 15°, indicating the amorphous properties of the matrix material [41].

### 3.3. Morphology Analysis

**Figure 6:**
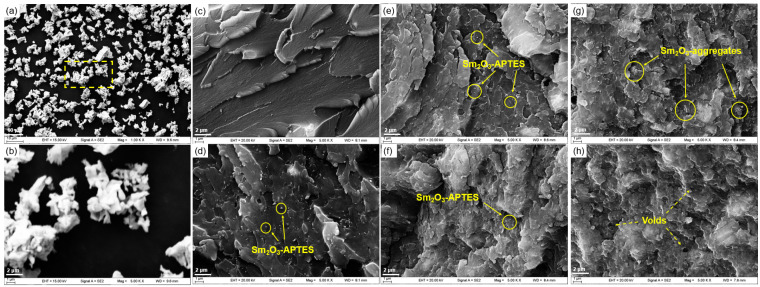
SEM images of Sm_2_O_3_-APTES particles and morphology of Sm_2_O_3_-APTES/AFG-90H: (**a**,**b**) Sm_2_O_3_-APTES particles; (**c**–**h**) Sm_2_O_3_-APTES content: 0, 5, 10, 15, 20, and 30 wt%.

**Figure 7:**
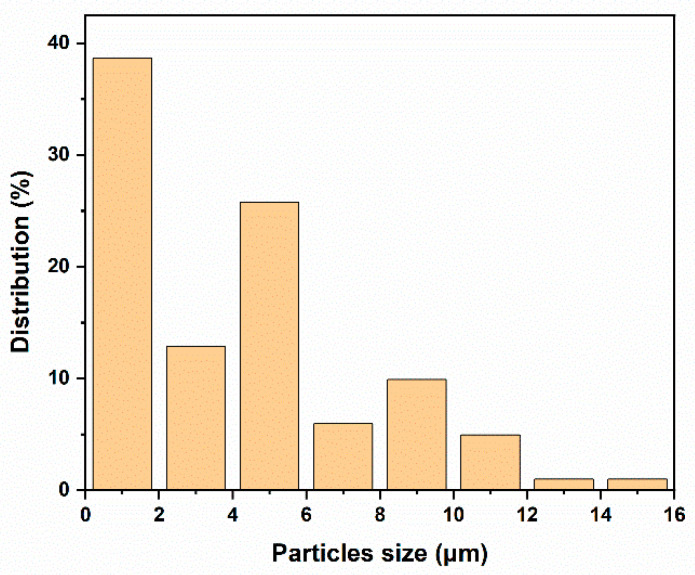
Size distribution of Sm_2_O_3_-APTES powders.

The morphology of the fractured surface of the Sm_2_O_3_-APTES/AFG-90H composite was shown in Figure 6. The particles show an irregular polyhedron shape with fibrous protrusions and slight agglomeration (as shown in Figure 6a,b). The section of pure AFG-90H resin was smooth (as shown in Figure 6c,d). With the increase in Sm_2_O_3_-APTES content, the fractured surface changed from smooth to rough. When the Sm_2_O_3_-APTES content was less than 20 wt%, it was uniformly dispersed into the matrix resin and there were fewer interfacial voids (as shown in Figure 6e,f). At a content above 20 wt%, the particles began to agglomerate (as shown in Figure 6g,h) due to the increase in the probability of the interconnection of active free groups on the particle surface. Because the interfacial energy of the formation of aggregates between particles was less than the binding energy of particles and the matrix molecular chain, there was no obvious separation of aggregates from the matrix, indicating that the adhesion with the resin matrix was good, because the free radicals on the surface of the filler helped to strengthen the interfacial bonding energy between Sm_2_O_3_-APTES and AFG-90H. However, when the Sm_2_O_3_-APTES content was 30 wt%, an increase in the number of pores in the matrix was observed. Therefore, the extension of dispersion for the Sm_2_O_3_-APTES particles in the matrix should be optimized during the preparation process. In addition, the particle size distribution is shown in Figure 7. The average size of Sm_2_O_3_-APTES was approximately 1.16 μm. Sm_2_O_3_-APTES particles of micrometer size can have a pinning effect on the epoxy resin matrix, which will be described in detail in Section 3.5.

### 3.4. Thermal Performance

**Figure 8:**
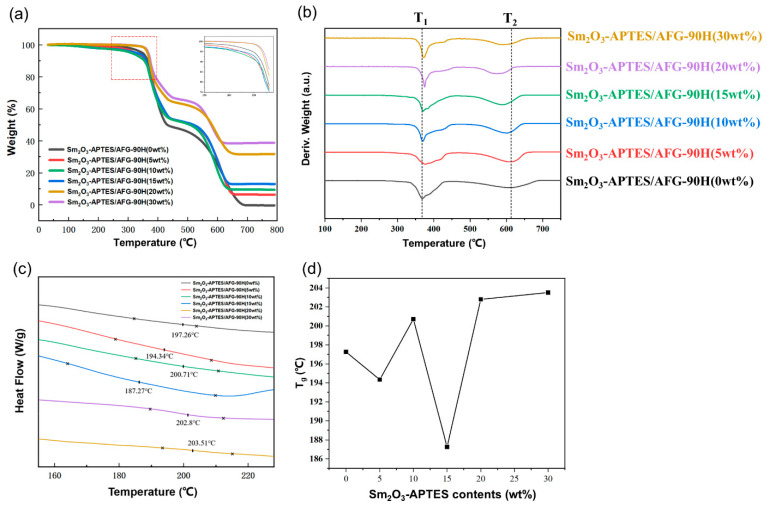
Thermal performance curves: (**a**) TG; (**b**) DTG; (**c**) DSC and (**d**) T_g_ of Sm_2_O_3_-APTES/AFG-90H.

The TG, DTG and DSC thermal analysis curves of Sm_2_O_3_-APTES/AFG-90H are shown in Figure 8. The mass loss temperatures of the TG curve are listed in Table 2, where T_5_ represents the temperature with a mass loss of 5 wt%, so for T_10_, T_50_ and T_max_.

As shown in Figure 8a, the two-stage decomposition process was found in all specimens, and these stages showed the conformation of the thermal decomposition trend for AFG-90H matrix. In the first stage, the thermal weight loss occurred at approximately 350 °C and the weight loss step continued to 430 °C. This decomposition reaction is attributed to the decomposition reaction of the epoxy network [42,43]. Owing to the existence of rigid structures (i.e., a benzene ring and an epoxy group) in the resin matrix, further decomposition of the composite was hindered. So, the curve remained flat in the temperature range from 430 to 530 °C [44]. In the second stage, the thermo-gravimetric range was from 530 to 650 °C. The polymer chain was completely decomposed and then the Sm_2_O_3_ was entirely separated from the matrix. The mass loss temperature was listed in Table 2, T_5_ followed a downward trend when the Sm_2_O_3_-APTES content increased from 0 to 10 wt% and an upward trend when the content increased from 15 to 30 wt%, while the same was observed for T_10_. When the Sm_2_O_3_-APTES content was 30 wt%, T_5_ and T_10_ increased by 5.4% and 3.6%, respectively, compared with that of the pure AFG-90H matrix. Microparticles increased physical crosslinking points and enhanced interfacial interactions. They play a positive role in inhibiting the initial decomposition [45].

The above results show that the agglomeration occurring with a high content of Sm_2_O_3_-APTES had a strong blocking effect on the thermal movement of the AFG-90H molecular chain, such as the weak chemical interaction between Sm_2_O_3_-APTES and AFG-90H. However, the maximum decomposition temperature T_max_ of Sm_2_O_3-_APTES/AFG-90H decreased compared with that of pure AFG-90H. The decreased maximum decomposition temperature T_2_ can also be seen in Figure 8b. In addition, T_g_ at the same heating rate as TG was calculated through the DSC curve (as shown in Figure 8c). The T_g_ of Sm_2_O_3_-APTES/AFG-90H with a low filler content (≤15 wt%) was irregular. This was due to the weak chemical force between the matrix molecular chains and Sm_2_O_3_-APTES with a low content. When the Sm_2_O_3_-APTES content was 30 wt%, T_g_ increased by 3.16% compared with that of pure AFG-90H.

The trend of T_g_ of Sm_2_O_3_-APTES/AFG-90H is shown in Figure 8d. Generally, T_g_ of polymer composites is affected by molecular weight, cross-linking density and particle-matrix interface area [46]. The trend of T_g_ vs. temperature could be due to the combined effects of two opposite factors [47]. On the one hand, the strong interfacial interaction between AFG-90H matrix and Sm_2_O_3_-APTES fillers could partially limit the motions of polymer chain segments and delay the occurrence of relaxation behavior at the glass transition region. This will reduce the flexibility of epoxy chain at high temperature and then lead to an increase in T_g_. On the other hand, the addition of fillers could cause difficulty for the composites to achieve the same level of curing as the neat AFG-90H, and thus the cross-liking density decreases and leads to a decrease in T_g_. In this work, when the content of Sm_2_O_3_-APTES is more than 20 wt%, the free volume of the resin decreases and the thermal movement of the chain end is limited by entangled Sm_2_O_3_-APTES agglomeration. Similar results can be found in Refs. [48,49]. The polymer chains need to absorb more energy to overcome the energy barrier, which leads to an increase in T_g_. Conversely, T_g_ changes irregularly due to the interaction of the above two factors. Ref. [50] showed similar results on the polymer incorporated with APTES-modified particles.

### 3.5. Mechanical Properties of Sm_2_O_3_-APTES/AFG-90H

**Figure 9:**
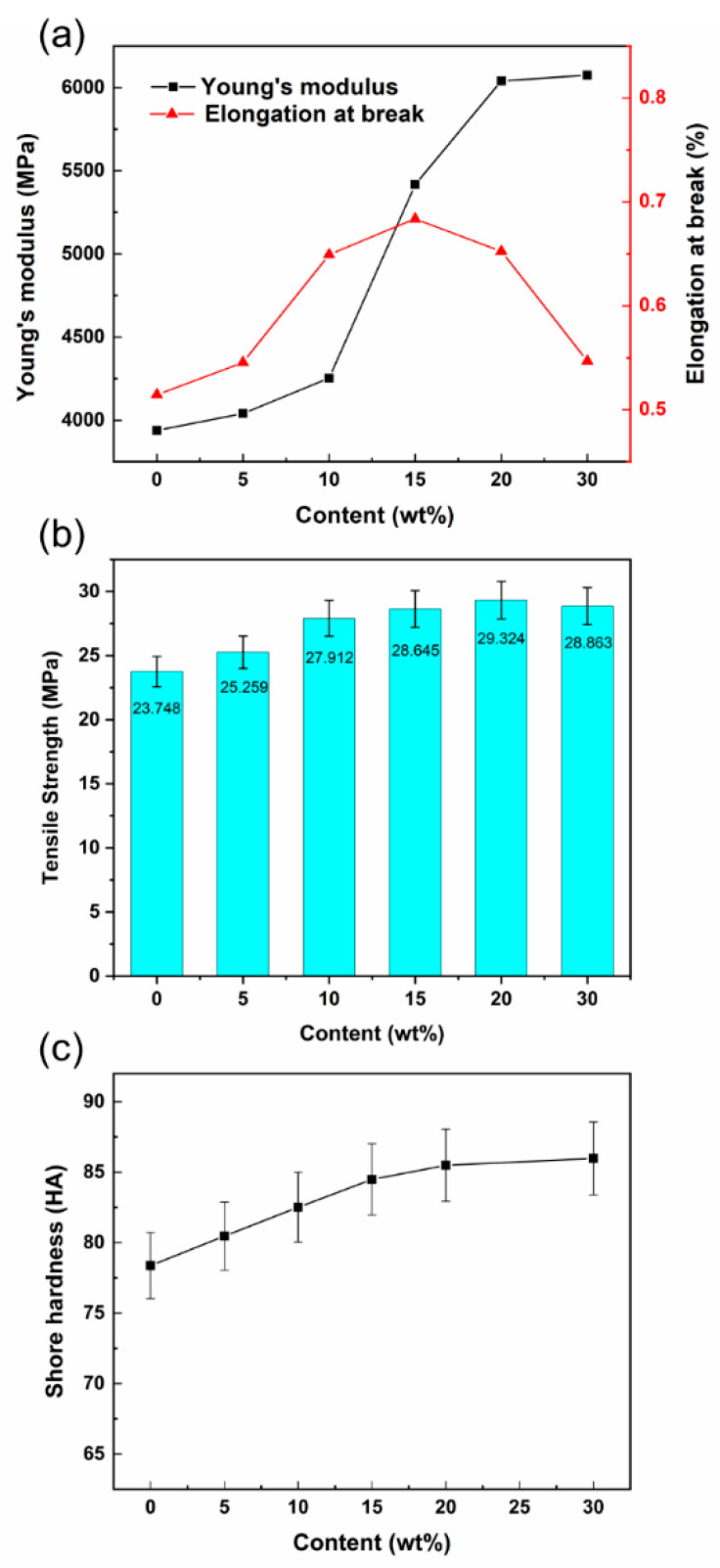
Mechanical properties of Sm_2_O_3_-APTES/AFG-90H: (**a**) Young’s modulus and elongation at break; (**b**) tensile strength; (**c**) Shore hardness.

The mechanical properties of Sm_2_O_3_-APTES/AFG-90H are shown in Figure 9, including Young’s modulus, elongation at break, tensile strength and Shore hardness. It is clear that the Young’s modulus of Sm_2_O_3_-APTES/AFG-90H composites increased with an increase in Sm_2_O_3_-APTES content (as shown in Figure 9a). When the content of Sm_2_O_3_-APTES was 0–5 wt%, the Young’s modulus increased slowly due to the good interfacial bonding between Sm_2_O_3_-APTES and the AFG-90H chains. When its content was 10–20 wt%, the Young’s modulus growth increased faster with uniform dispersion of Sm_2_O_3_-APTES and good interface compatibility, which synergistically improves the modulus of composite materials. When its content was 30 wt%, the increased probability of Sm_2_O_3_ agglomeration could destroy the mechanical properties of the composite material, which was shown in Figure 9b. From the perspective of Shore hardness, the surface hardness of the material gradually increases with Sm_2_O_3_-APTES and finally reaches a saturation value (as shown in Figure 9c). The texture of the composite material is brittle and harder, indicating that Sm_2_O_3_-APTES can slightly increase the surface hardness of AFG-90H. Composite stiffness significantly depended on the Sm_2_O_3_-APTES particle content but not the particle/matrix adhesion, since the fillers had a much higher modulus than the matrix [51].

**Figure 10:**
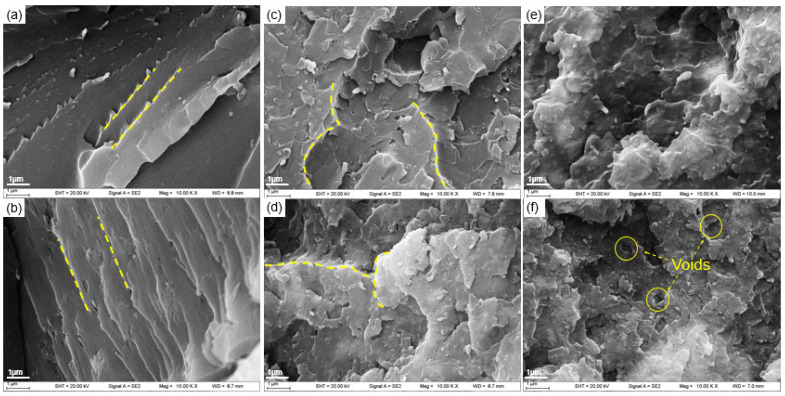
SEM images of tensile fracture surfaces for composites with different Sm_2_O_3_-APTES contents: (**a**–**f**): 0, 5, 10, 15, 20 and 30 wt%.

**Figure 11:**
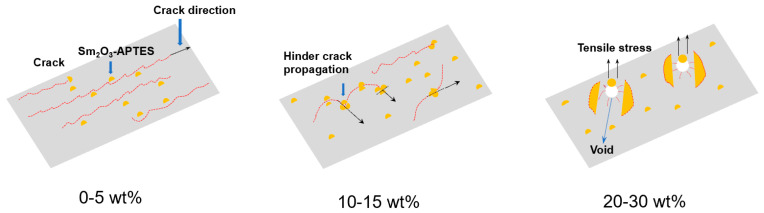
Mechanism diagram of Sm_2_O_3_-APTES improving mechanical properties of composites.

One criterion for judging the toughness of polymer materials is the elongation at break. The elongation at break of the composite reached a peak of 0.68%, and T_g_ was the lowest when the Sm_2_O_3_-APTES content was 15 wt% in this study. In order to further study the effect and mechanism of Sm_2_O_3_-APTES on the toughness of the composite material, the microscopic morphology of the tensile cross-section of the composite material was observed, as shown in Figure 10. When the Sm_2_O_3_-APTES content is 0–5 wt%, the fracture cross-section of the composites is neat and there is a single crack direction. Typical brittle fracture characteristics are shown in Figure 10a,b. When the content of Sm_2_O_3_-APTES is 5 wt%, a small amount is distributed in the AFG-90H matrix, which has little effect on the cracks and the good interface adhesion between Sm_2_O_3_-APTES and the matrix increases the material’s toughness. When the content of Sm_2_O_3_-APTES is 10–15 wt%, the cracks are deflected, as shown by the yellow curve in Figure 10c,d, indicating that Sm_2_O_3_-APTES can be pinned in the matrix to prevent the cracks from propagating along the original direction among the contents [52]. The delamination section appears because the stress gradually concentrates as the content increases. When its content is 20–30 wt%, agglomerates began to appear, the delamination phenomenon caused by stress concentration is more obvious and the compatibility of the agglomerated part and the matrix becomes poor. Therefore, part of the agglomerate comes out of the AFG-90H matrix when the tensile stress is loaded, causing holes and generating surrounding micro-cracks [53]. The rapid propagation of micro-cracks reduces the toughness of the matrix. The mechanism was analyzed based on the SEM images, and its diagram is shown in Figure 11. At a low Sm_2_O_3_-APTES content (≤15 wt%), the crosslinking of particles and molecular chain can effectively disperse the stress. At a high Sm_2_O_3_-APTES content (>15 wt%), the tensile stress of the matrix to the fillers is higher than its interfacial adhesion force. Therefore, the agglomerations have interfacial debonding and holes [54,55]. A large amount of local plastic deformation is exhibited around the equator of the particles. Hence, the overall fracture toughness is reduced.

### 3.6. Neutronics Simulation

**Figure 12:**
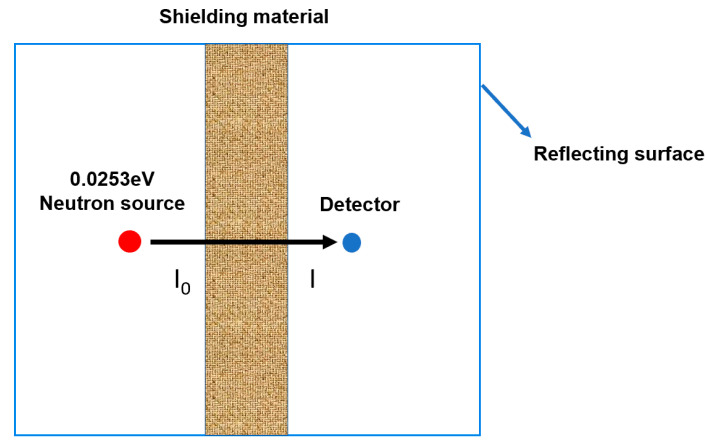
Schematic diagram of one-dimensional neutron shielding model.

Monte Carlo methods are widely used in nuclear engineering [56,57]. In this work, the neutron shielding performance of Sm_2_O_3_/AFG-90H composite is simulated by the SuperMC software. The one-dimensional physical model adopted for the neutron shielding performance of the shielding material is shown in Figure 12. The simulation was performed in fixed neutron source mode. An isotropic thermal neutron point source with an average energy of 0.0253 eV was used and the fluence was 10^6^ n/(cm^2^·s). The distance between the neutron source and the shielding material was 10 cm. The length perpendicular to the incident direction was defined as infinite, based on the assumption that all elements in the shielding material were uniformly dispersed. At least three measurements with analog point neutron detectors were performed. The neutron shielding performance of the material was statistically evaluated by neutron permeability I/I_0_. The following equation was used for I/I_0_ [58]:

η = I/I_0_ = Ae^−Σd^ + b

where η is the transmittance, I and I_0_ are the intensities of the transmitted and incident neutron fluences (n/cm^2^), A is the accumulation factor, d is the thickness of shielding material (cm), Σ is the total macro-cross-section (cm^−1^) of all elements in the composite, and b is the background count. The secondary γ doses generated by the shielding material after capturing the thermal neutrons were recorded by the detector, and the flux was converted into dose through the dose conversion factor [59] to assess the level of gamma radiation. In addition, the Sm isotopic abundance and average thermal neutron absorption cross-section are listed in Table 3.

**Figure 13:**
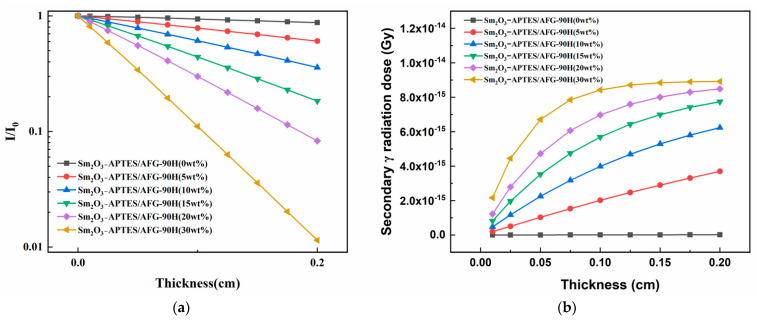
Shielding performance of Sm_2_O_3_-APTES/AFG-90H: (**a**) neutron permeability (I/I_0_); (**b**) secondary γ dose.

The neutron shielding performance of Sm_2_O_3_-APTES/AFG-90H is shown in Figure 13. It is clear that the value of the transmittance η decreased linearly with the increase in shielding material thickness and decreased with the increase in Sm_2_O_3_-APTES content (as shown in Figure 13a), which indicated that the Sm element had good thermal neutron absorption performance. In the case of theoretical calculation, the η value for 30 wt% Sm_2_O_3_-APTES/AFG-90H with a thickness of 0.2 cm is 98.86%, which is higher than that of the AFG-90H matrix. The secondary γ dose released by the shielding materials after absorbing thermal neutrons is shown in Figure 13b. The secondary γ dose produced by the composites after absorbing thermal neutrons was less than 10^−14^ Gy. With the increase in Sm_2_O_3_ content, the trend of the γ dose gradually transforms from a linear increase to a logarithmic increase. This indicates that the secondary γ dose generated by the composite material absorbing thermal neutrons will reach a saturation value with the increasing content of Sm_2_O_3_. The secondary γ at this dose level will be scattered in the environment without causing adverse damage to the human body or the environment.

Moreover, Table 4 summarizes the physical properties of epoxy-based neutron shielding materials, including the decomposition temperatures of different mass loss, tensile strength and neutron permeability of different epoxy-based composites [60,61,62]. It shows that Sm_2_O_3_-APTES/AFG-90H possessed better thermal stability and neutron shielding properties compared with other epoxy-based composites. The Sm_2_O_3_-APTES/AFG-90H with 20–25 wt% Sm_2_O_3_-APTES had good neutron shielding properties and the potential to be used as neutron shielding materials for advanced nuclear system.

## 4. Conclusions

In this paper, a new type of high-performance epoxy resin AFG-90H and rare earth oxide Sm_2_O_3_ composites was prepared. The effects of Sm_2_O_3_-APTES content on thermal properties, mechanical properties and neutron shielding properties were studied. The mechanism of its effect on mechanical properties was deeply studied. The main conclusions are as follows:

(1) The uniform dispersion of Sm_2_O_3_ particles and good interface compatibility improved the strength and stiffness of Sm_2_O_3_-APTES/AFG-90H composites. When the Sm_2_O_3_-APTES content is 15 wt%, the composites have higher fracture toughness.
(2) The Sm_2_O_3_-APTES hindered the thermal motion of molecular chains and improved the thermal stability of the composite at 340–380 °C. Simultaneously, the glass transition temperature T_g_ of the composites is slightly increased.
(3) The shielding simulation showed that 30 wt% Sm_2_O_3_-APTES/AFG-90H had a higher neutron shielding performance than the AFG-90H matrix. After capturing neutrons, the secondary γ dose was 8.5 × 10^−15^ Gy.
(4) The appropriate range of Sm_2_O_3_-APTES content in the epoxy matrix is 20–25 wt% to obtain higher properties.

In future work, the properties of Sm_2_O_3_-APTES/AFG-90H under neutron irradiation will be studied. The results indicate that the polymer composites have the potential for application in radiation protection.

## Figures and Tables

**Table 1:** Basic properties of AFG-90H polymer.

Physical Properties	Value
Mw	556
Mn	317
Density (g/cm^3^)	1.314
Cross-linking density	2179
Viscosity (mPa·s@25 °C)	1540
Epoxy equivalent (g/mol)	105

**Table 2:** TG data for Sm_2_O_3_-APTES/AFG-90H (℃).

Sm_2_O_3_-APTES Content (wt%)	T_5_	T_10_	T_50_	T_max_
0	347.7	361.4	431.5	693.7
5	325.2	357.1	514.4	659.9
10	309.6	354.5	506.7	644.9
15	327.3	360.8	519.7	646.2
20	364.5	371.1	581.3	662.4
30	367.5	374.4	578.3	623.7

**Table 3:** The isotopic composition and thermal neutron cross-section of natural samarium.

Isotope SAm	Atomic Abundance (%)	σ_A_ (b)	σ_A,w_ (b)
S144m	3.1	0.7	0.02
S147m	15.1	57	8.61
S148m	11.3	2.4	0.27
S149m	13.9	42,080	5849.12
S150m	7.4	104	7.70
S152m	26.6	206	54.80
S154m	22.6	8.4	1.90

**Table 4:** The decomposition temperature of different mass loss, tensile strength, neutron permeability of epoxy-based neutron shielding materials.

Shielding Materials	Decomposition Temperature (°C)	Tensile Strength (MPa)	Neutron Permeability (%)	Reference No.
B_4_C (5%)/epoxy resin	218 (5% mass loss)	32	56 (0.2 cm thickness)	[60]
Pb/B/GO/epoxy resin	332 (5% mass loss)	/	/	[61]
Gd (3%)/epoxy resin	342 (20% mass loss)	46	66 (0.5 cm thickness)	[62]
Sm_2_O_3_-APTES (20%)/AFG-90H	364 (5% mass loss)	29	82 (0.2 cm thickness)	Our results

## Data Availability

Not applicable.
